# The feasibility of teleconsultations in unplanned primary care: an intervention study in Belgium, 2021

**DOI:** 10.1186/s13690-023-01058-7

**Published:** 2023-03-24

**Authors:** Sarah Hertens, Chris Van der Mullen, Birgitte Schoenmakers

**Affiliations:** 1 Department of General Practice and Chronic Care, VUB, Laarbeeklaan 103, Brussel (Jette), 1090 Belgium; 2 Department of Public Health and Primary Care, KU Leuven, KU Leuven, Kapucijnenvoer 7, box 7001, Leuven, 3000 Belgium

**Keywords:** Primary care, Unplanned care, Triage, Teleconsultation

## Abstract

**Introduction:**

Since 2000, an increasing misuse of emergency services in Belgium was noticed. In 2015, a multidisciplinary task-force designed a triage system. Trained operators and integrated triage protocols were installed in a call center for life-threatening and non-life-threatening care needs. Teleconsultations by telephone find their way to planned care and are well studied in this context. Also unplanned care might benefit from telephone-consultations.

**Method:**

This intervention study investigated the feasibility of teleconsultations in unplanned care according to medical doctors. They were present at the call center during the weekend and on public holidays in the period of April 17, 2021 to November 21, 2021. Their task was to call patients who had contacted the call center to perform a teleconsultation, without interfering with regular care.

**Results:**

21 triage doctors participated in the study, they completed 59 surveys and conducted 551 teleconsultations. They perceived the quality of the consultations as good with an average score of 82.85 out of 100 on the sliding scale. The doctors gave an average score of 72.40 for the level of certainty for diagnosis and treatment. For 415 consultations, triage doctors judged that the consultation would gain certainty if followed by a physical examination. Video was mainly considered to be valuable in psychiatric problems, allergic reactions and skin problems.

**Discussion:**

This study showed that teleconsultations are feasible in unplanned care. Videos add value in particular cases. Only few barriers are reported in terms of communication, technology and equipment.

**Conclusion:**

Teleconsultations in unplanned primary care could be performed with a high quality and a sufficient level of certainty. The willingness to conduct teleconsultations in unplanned care is high. It would be useful in a future study to investigate the feasibility, obstacles and needs for implementation of video consultations as they may differ from teleconsultations.

## Introduction

At the beginning of this century, an increasing misuse of emergency services in Belgium was noticed. The Federal Knowledge Center for Healthcare (KCE) conducted a study on this in 2005 and 2016 on behalf of the Belgian Government [1]. They formulated the need for a clear description of the improper use of these services and the need for the development of a triage system to guide patients with a care need to the appropriate care provider [2].

In 2015, a multidisciplinary task-force from the Leuven-Tienen region joined to sign agreements between the out-of-hours GP (General practitioner)-venture and the hospital emergency services addressing unplanned care provision. Unplanned care includes acute, incidental, and unexpected patient care needs that require prompt medical assessment. The task-force designed a telephone triage system for this unplanned care and suggested using uniform integrated protocols for both life-threatening and non-life-threatening care [3].

The European call number 112 is intended for life-threatening unplanned care questions. In addition to this number, the call number 1733 is rolled out since October 2017 for non-life-threatening unplanned care needs in Belgium [2]. Both the calls to the 112 and the 1733 telephone numbers are answered by operators of the joint 112/1733 emergency center. These operators triage the patients according to the level of the care need and refer them to an appropriate level of care. They use uniform integrated protocols developed by the above-mentioned task force. These integrated medical triage protocols 1733 − 112 are written down in the Belgian Manual for Medical Regulation [4, 5].

Each call to 112/1733 for a medical care request is assigned by an operator using the triage protocols to a level of severity that corresponds to the appropriate type of regulation. Regulatory resources are resources that answer patient care needs. The following resources are currently available in Belgium: Mobile Emergency Group (MUG), Paramedical Intervention Team (PIT), ambulance, urgent GP (a home visit within 2 h after the call), non-urgent GP (a consultation in the guard post or a home visit with a maximum delay of 12 h after the call) and postponement of care (whereby follow up is referred to planned care). Postponement of care means a delay of a maximum 12 h in case the complaints do not disappear. Patients are always advised to call back if the complaints worsen [2, 3].

All current means of care regulation involve face-to-face contact between the patient and the healthcare provider. However, global digitization is increasingly finding its way into the clinical practice. The Covid-19 pandemic accelerated this trend. Keeping distance became the norm, with tele- and video consultations entering medical practices [6].

In 2019, the National Order of Doctors issued an opinion stating that a remote consultation does not have the same accuracy as a traditional face-to-face consultation and that teleconsultation does not offer the same safety for diagnosis and treatment. Teleconsultations are in this context defined as a telephone contact between doctor and patient. Therefore, they concluded that a teleconsultation could only replace the traditional consultation in case of a particular situation.

During the COVID19-pandemic, the National Order stated that this is such a particular situation that justifies teleconsultations defined as a telephone consultation. The Order also stipulated that doctors at least should understand the patient’s medical antecedents and care need by taking a careful and complete history if they do not have access to the patient’s medical record [7]. A report from the KCE showed that there is no evidence that teleconsultations are equal or better than face-to-face consultations or that they have a negative effect on patients’ health [8].

This study examines whether, according to medical doctors, teleconsultations (telephone contact) are feasible in unplanned primary care as additional means of regulation and under which conditions these teleconsultations should take place.

## Method

### Study design

This study includes an intervention. The outcome measure is the feasibility of teleconsultations in unplanned care in a primary care setting. Feasibility was defined as an assessment by the triage doctors of certainty, obstacles and conditions with regard to conducting teleconsultations.

In this study design, medical doctors were present at the 112/1733 emergency center in the province of Flemish Brabant during the weekend and on public holidays (24/24) in the period of April 17, 2021 to November 21, 2021. These doctors are further on referred to as triage doctors. Their task was to immediately call back patients who had contacted the 1733 number to evaluate the feasibility of teleconsultations, without interfering with regular care. Regular care was not interrupted and all calls were regulated as prescribed.

### Recruitment, inclusion and exclusion criteria

Only general practitioners, emergency doctors (Certificate Acute Medicine or Advanced Masters in emergency medicine), residents in general practice (HAIOs) and hospital residents in emergency medicine (ASOs) were included as triage doctors. All doctors had to be active in the Leuven-Tienen-Mechelen region. This region is traditionally the pilot region for investigations related to the 1733-unplanned care number. This region has the required expertise for research at an operational, academic level (Academic Center for General Practice KU Leuven) and organizational level (Adjunct director Dr Marc Gellens and co-supervisor Dr Chris Van der Mullen).

For the recruitment of doctors, an e-mail with information about the study was sent to the presidents of the GP networks and to the heads of department of the emergency services. On January 15 and 22, 2021 an information session about the research was held for those interested. After this information session, attendees could declare their interest in this study.

Patients were recruited to participate in the study at the moment of their call to the 1733 number and were called back immediately after their consent.

The following inclusion criteria were used: older than 16 years, Dutch speaking and mentally and physically capable to conduct a teleconsultation as assessed by the triage doctor. Additionally, for children under the age of 16, at least one of the primary caregivers of the child (usually the parents) had to be present near the child. Only first calls to 1733 for a particular patient’s medical complaint were included.

Patients were eligible for inclusion if their medical complaint was triaged by the operator within severity level 7 (= not urgent GP). At this level, the patient is referred to the out-of-hours medical center or a non-urgent home visit is planned. Every reason for encounter (patients’ complaint translated by the triage protocol) was eligible.

An additional condition was that the patient had not yet been examined by the doctor on duty in the out-of-hours medical center or on a home visit.

All patients who met the inclusion criteria could be called by the triage doctors. These triage doctors introduced themselves clearly, explained the purpose, the design, the implications of this teleconsultation and confirmed the continuation of regular care. Only if the patients fully understood and explicitly agreed to participate, they were included in the study.

The patients were informed by the triage doctors that they were not forced to participate in the study and that the teleconsultation with the triage doctor could be terminated at any time upon their request.

### Intervention

When a patient called the 1733 number, a regular operator of the 112/1733 emergency center answered the call. This operator handled the call as usual using the federal medical triage protocols 112–1733 version 4.01. If the patient met the inclusion criteria and the call was triaged by the operator within severity level 7, the patient was asked permission to be called back by a triage doctor of the study. The patients were called by the triage doctors before the actual consultation in the out-of-hours medical center or the home visit had taken place.

The triage doctors questioned the medical history of the patients and their current complaints. This medical information was not saved into a patient file and only used to assess the feasibility of a teleconsultation. The triage doctors did not communicate a diagnosis to the patients and did not provide medical advice. Each patient was still examined and treated by the on-call doctor during a consultation in the medical center or during a home visit but this information was not available for study purposes.

Each teleconsultation took maximum ten minutes. There was no template to perform the teleconsultations. The triage doctors used the gear of the 112/1733 Emergency Central to guarantee a stable quality of the telephone interventions. The teleconsultations were recorded and stored in ASTRID’s database in compliance with the General Data Protection Regulation (GDPR) and used for study purposes only.

With each shift in the 112/1733 emergency center in Flemish Brabant, the triage doctors completed a digital questionnaire via the QualtricsTM-KU Leuven program on a study laptop. Each doctor was free to fulfill one or more shifts in the call center during the study weekends and days. A doctor can therefore have completed several questionnaires.

The questionnaire addressed the feasibility of teleconsultations within unplanned care. The questionnaire addressed the self-assessed certainty with which the consultations were completed, the obstacles of the teleconsultations and the conditions for making teleconsultations more efficient. Answers were indicated on a sliding scale from 1 to 100 (low to high performant or agreement), by yes/no and completed with free text fields. No personal or medical patient data were collected in this questionnaire. (attachment 1)

### Data analysis

The primary outcome measure is the feasibility of the teleconsultations measured as certainty of completion of consultations, obstacles and conditions for implementation. The background variables are gender, medical specialty and work experience of the participating doctors.

The dataset was downloaded from the QualtricsTM-KU Leuven program in the form of a Microsoft® Excel file. The processing of the data was also carried out by means of the Microsoft® Excel program.

The background variables were reported using descriptive statistics. For the answers to the sliding scales and the yes/no questions, a univariate descriptive statistic was used.

*Ethical considerations*.

This study was approved per research question by the Medical Ethical Committee of the University Hospitals of the KU Leuven (MP017505 23/03/2021). Patients gave their explicit, verbal informed consent and no medical patient data were saved or used in the data analysis.

## Results

A total of 21 triage doctors participated in the study, including 13 general practitioners, 2 general practitioners-in-training (HAIOs), 2 emergency doctors and 4 assistants-in-training (ASOs) in emergency medicine (Table 1).

**Table 1:** Number of teleconsultations per specialty and per years of working experience/training

Discipline	Working epxperience/traning	Number of teleconsulations, n (%)
Huisarts		
	< 5 years	153 (28,65)
	5–10 years	88 (16,48)
	10–20 years	0 (0,00)
	20–30 years	67 (12,55)
	> 30 years	4 (0,75)
	**Total**	**312 (58,43)**
**HAIO**		
	Year 1	0 (0,00)
	Year 2	39 (7,30)
	Year 3	11 (2,06)
	**Total**	**50 (9,36)**
**Emergency doctor (1) certified in emergency care**		
	< 5 years	0 (0,00)
	5–10 years	0 (0,00)
	10–20 years	35 (6,55)
	20–30 years	17 (3,18)
	> 30 years	0 (0,00)
	**Total**	**52 (9,74)**
**Emergency doctor (2) master in emergency care**		
	< 5 years	7 (1,31)
	5–10 years	0 (0,00)
	10–20 years	0 (0,00)
	20–30 years	0 (0,00)
	> 30 years	10 (1,87)
	**Total**	**17 (3,18)**
**ASO Emergency**		
	Year 1	33 (6,18)
	Year 2	51 (9,55)
	Year 3	19 (3,56)
	Year 4	0 (0,00)
	Year 5	0 (0,00)
	Year 6	0 (0,00)
	**Total**	**103 (19,29)**

Together they completed 59 surveys and conducted 551 teleconsultations. This is an average of 9.34 teleconsultations per survey (and therefore per shift in the emergency center). The median number of teleconsultations per survey is also 9. Of the 551 teleconsultations performed, 17 were excluded from analysis (3.18%). In 11 of these teleconsultations, the patient had already been examined by the on-call GP. Two teleconsultations concerned a non-Dutch-speaking patient. In three teleconsultations the patient refused further participation and in one case the patient was unreachable (voicemail).

### Background variables

Of the 534 remaining teleconsultations, the majority was performed by a GP (312, 58.43%). A total of 153 (28.65%) of the teleconsultations were performed by a young GP with a maximum of 5 years of work experience. Almost 1/5 of the teleconsultations was performed by an emergency doctor (103, 19.29%) (Table 1).

The initial call to the 1733 number was triaged to the regulatory means “Consultation in the out-of-hours medical center” (83.33%) in 445 of the teleconsultations.

The protocol “080: Suspicion of COVID-19” was most often chosen by the operators in the initial calls (95, 17.79%). The protocols “064: Nose-Throat-Ear and Dental Problems” and “012: Non-traumatic abdominal burden” follow in 2nd and 3rd place with 11.80% and 8.43% of the teleconsultations respectively (Table 2).

**Table 2:** Overview of the protocols chosen for the initial calls of the teleconsultations (n = 534), level of certainty and added value of physical examination on a scale from 1-100 poor to high

Protocol	Times chosen n (%)	Certainty level (average)	Added value physcial examination (average)
001: traffic accident	1 (0,19)	70,00	100,00
002: aggression –fight- sexual violation	2 (0,37)	63,00	100,00
006: burns - abrasions	1 (0,19)	39,00	64,00
010: Breathing difficulties	14 (2,62)	77,85	83,60
011: chest pain	3 (0,56)	98,33	55,00
012: non traumatic abdominal pain	45 (8,43)	69,36	60,78
013: non traumatic back pain	32 (5,99)	67,06	66,87
016: pregnancy-delivery	1 (0,19)	100,00	0,00
017: non traumatic blood loss	3 (0,56)	68,00	50,00
019: unconsciousness - Coma - Syncope	2 (0,37)	24,00	91,00
025: non traumatic headache	9 (1,69)	57,67	67,00
026: unwell for no clear reason	20 (3,75)	78,47	67,22
027: bites	7 (1,31)	58,14	58,14
031: psychiatric problems	1 (0,19)	92,00	/*
032: allergic reactions	21 (3,93)	80,67	72,70
033: trauma	37 (6,93)	77,03	81,60
034: brain injury	2 (0,37)	65,50	89,00
039: Cardiac trauma (not chest pain)	6 (1,12)	79,80	78,17
059: diziness - Vertigo	6 (1,12)	52,33	79,80
060: skinproblems	35 (6,55)	66,77	74,97
061: limb cold or warm (not trauma)	32 (5,99)	67,47	72,06
063: eye problems	18 (3,37)	66,28	69,00
064: nose-throat-ear-dental problems	63 (11,80)	71,76	70,52
065: sudden deafness	1 (0,19)	61,00	89,00
066: post-operative problem	6 (1,12)	83,17	77,75
068: urogenital problem	24 (4,49)	75,71	56,62
069: wounds (light)	16 (3,00)	65,93	65,13
071: sick child > 3 months and < 15 y with abdominal pain	2 (0,37)	91,00	91,00
072: sick child > 3 months and < 15 y with fever	11 (2,06)	84,10	86,30
073: sick child > 3 months and < 15 y with airway infection	6 (1,12)	75,00	70,00
074: palliative patient	1 (0,19)	90,00	90,00
080: suspected COVID-19	95 (17,79)	77,81	53,47
No protocol chosen	11 (2,06)	63,30	73,50

In 72.41% of the completed surveys the doctor indicated experience with teleconsultations (Table 3). However, some doctors completed multiple shifts in the emergency center, completed multiple questionnaires and gained experience in teleconsultations.

**Table 3:** Experience of the triage doctors with tele and/or video consultations in the surveys (n = 58)

Experience with tele- or video consultations	Number of surveys n (%)
No experience	14 (24,14)
Experience with teleconsultations	42 (72,41)
Experience with tele- and video consultations	2 (3,45)

### Teleconsultations

The doctors perceived the quality of the consultations as good with an average score of 82.85 out of 100 on the sliding scales (median of 90). The quality of the patient contact was also positively evaluated with an average score of 85.30 (median of 92).

The doctors rather did not miss the face-to-face contact with the patient showing an average score of 38.41 (median of 29) (Table 4). Doctors did not experience many communication problems, with an average score of 18.14 (median of 2) and they mainly named communication barriers (low health literacy) and hetero-anamneses (Table 4).

**Table 4:** Assessment of teleconsultations by triage doctors according to quality, certainty, obstacles and willingness to carry out teleconsultations in the future (scale from 1-100 poor to high or disagree to agree)

Questions	Average	Median
Quality of consult?	82,85	90
Quality of patient contact?	85,30	92
Missed face to face contact?	38,41	29
Communication problems with patient?	18,14	2
Technical problems?	10,55	1
Level of certainty?	72,40	81
Added value of physical examination?	67,62	79,5
Added value of video connection?	39,97	32
Prepared to use teleconsultations in the future?	80,85	90

Technical issues were rarely reported with an average score of 10.55 (median of 1) (Table 4). Nearly all reported technical issues related to a poor or unstable connection.

In 364 (68.29%) cases, the triage doctors believed that the teleconsultation was not sufficient to provide care. A teleconsultation was found sufficient to exclude a serious condition or to make a differential diagnosis (51.2% and 33.73%), respectively. A teleconsultation was not sufficient for specifying a diagnosis and assessing the severity of the care need (resp 21.13% and 28.45%).

In 33.24% of the answers to the question why teleconsultations are not sufficient were classified as “Unclear Answer” and mostly only stating that ‘a physical examination is necessary’. In the majority of the teleconsultations that were classified as ‘not sufficient’, a video connection was not considered as an added value according to the triage doctors (n = 278, 76.37%).

The extent to which a physical examination could have contributed to the diagnosis was also determined per protocol (Table 2). For the protocols “001: Traffic accident”, “002: Aggression – Fight – Rape”, “019: Unconsciousness – Coma – Syncope”, “071: Sick child > 3 months and < 15 years with abdominal pain” and “074: Palliative patient ” triage doctors scored on average above 90.

Considered per triage protocol, it appeared that video was mainly considered to be valuable in psychiatric problems, allergic reactions and skin problems. Also for the protocols addressing eye problems, post-operative problems and non-traumatic blood loss the average scores referring to the added value of videos were are above 60 (Table 2). When asked “How could video have contributed?” the answers were mainly classified within the categories “Assessing the severity” and “Differential diagnostic” (resp 56.15% and 40.77%).

The doctors gave an average score of 72.40 for the level of certainty with which the consultation was completed (median of 81). To the question “To what extent could a physical examination have contributed to the diagnosis in this consultation?” the triage doctors gave an average score of 67.62 (median 79.5) (Table 4).

Doctors indicated 158 times (29.81% of all teleconsultations) that the consultation could gain certainty using a video connection. The extent to which video could have contributed to the diagnosis in the consultation was estimated to be relatively low with an average score of 39.97 (median of 32) (Table 5).

**Table 5:** The certainty of triage doctors with which the consultations were completed per discipline

Discipline (n = number of teleconsultations)	Average
GP (n = 312)	66,20
HAIO (n = 50)	93,14
Emergency doctor 1 (n = 52)	80,62
Emergency doctor 2 (n = 17)	80,88
ASO emergency (n = 103)	75,19

Legend: HAIO = GP in training, ASO = hospitals specialist in training

There are some differences to note between the different medical specialties (Table 5). HAIOs indicated a higher level of certainty than the other doctors with an average of 93.14 on the sliding scale. The GPs indicated the lowest level of certainty with 66.20 as the average score.

The degree of certainty of completion of the teleconsultations per protocol was also investigated (Table 2). Only two protocols were assigned a mean score of less than 50 (low certainty), namely protocol “006: Burns – Abrasions” and protocol “019: Unconsciousness – Coma – Syncope”. The highest scores were assigned to the protocols “016: Pregnancy - delivery”, “011: Chest pain” and “031: Psychiatric problems”, “071: Sick child > 3 months and < 15 years with abdominal burden” and “074: Palliative patient”.

For 415 (77.86%) consultations, triage doctors judged that the consultation would gain certainty if followed by a physical examination. In these cases, a physical examination could contribute to the differential diagnosis and to diagnostic certainty (48.49%). The assessment of the severity of care need was also frequently mentioned as a possible added value of a physical examination (34.34%).

The questionnaire surveyed the extent to which doctors were willing to use teleconsultations during the on-call service in the future. The triage doctors gave a mean score of 80.85 on whether they would use on-call teleconsultations in the future (Table 2).

For the implementation of teleconsultations during an on-call shift, triage doctors mainly raised the need for an algorithm for referencing a teleconsultation (47.03%). A video connection and the preparation of digital prescriptions and certificates were mentioned as additional needs (respectively 14.36 and 14.85%).

## Discussion

The aim of this study was to determine whether teleconsultations are feasible in unplanned primary care and which conditions are required to make it feasible. This study showed that teleconsultations can be performed with a good level of certainty and a high quality of patient contact, but under condition that clear triage protocols are available to define the nature of teleconsultations. The protocols addressing COVID-19, common colds in all varieties and abdominal pain were most applied. The possibility of creating and delivering medication prescriptions and certificates and the availability of a video connection are also indicated as important needs. Only few barriers are reported in terms of communication, technology and equipment.

The overall quality of the consultations was experienced as very good but not sufficient to provide care. The face-to-face contact with the patient was only moderately missed by the triage doctors. Communication and technical difficulties were in only few cases experienced. Mainly hetero-anamneses and miscommunication were reported as communication problems. These barriers are in agreement with other research addressing teleconsultations [9, 10].

The technical problems that were reported mainly concerned a bad connection which corresponds to the current research in this field [10]. It is not unthinkable that more or other communication or technical problems are reported during video consultations [10].

Doctors rated the teleconsultations as not sufficient to provide care but rather enough to rule out serious conditions. This is in line with the responsibility of the triage task of the operators and appeared to be to be an effective and safe approach in terms of patient related outcome [5].

The level of certainty with which the consultations were completed appeared to be sufficiently high. The very high degree of certainty reported by the HAIOs is striking but might be related to the higher level of confidence and experience these younger doctors have in distance or digital communication [11, 12]. This might suggest that the level of certainty is linked to digital skills and might be trained [13, 14]. We can also assume that since there was no regulation on teleconsultation before the COVID19-pandemic, triage doctors, and by extension all doctors, have more or less the same baseline skill level in teleconsultation.

In the majority of teleconsultations doctors indicated that a physical examination might have raised the level of certainty. Video was considered as less valuable to achieve this target. As expected, the contribution of video in the diagnosis was considered to be particularly higher for ‘visible complaints’ such as skin diseases but also for a supportive talk in case of psychiatric problems [8, 10, 15].

The level of certainty and the willingness to perform teleconsultations in the future also appeared to be high. Nevertheless, two third of the triage doctors indicated that teleconsultations were not sufficient to provide care. When closely exploring this statement, it appeared that the answers were rather vague and unclear (questioned as free text) and probably biased by a negative experience. We believe that this observation might be related to feelings of insecurity and a lack of confidence in digital skills [11, 13, 16]. On the other hand, teleconsultations might fill a particular gap in unplanned or emergency care by triaging patients for more effective workflows and to prevent from overcrowding in emergency departments [3, 5].

The willingness to conduct teleconsultations is high and remarkably higher among the HAIOs. Here also, HAIOs might be more familiar with teleconsultations because of their age and because the start of their residence overlapped with the COVID19 pandemic. They might be keener to learn and to perform consultation under any circumstances.

The level of certainty also varied per reason for encounter translated into a specific protocol. The protocol on burns and on problems of consciousness were related to a lower certainty. This might be inherent to the need of visual assessment of a wound and of the severity of the loss of consciousness.

There is a major need now to draw up a protocol with conditions and criteria defining the nature of teleconsultations for call takers to triage patients to the correct care level [5]. It appeared that some reasons for an unplanned care consultation required more than others a physical examination [8, 15].

Furthermore, the availability of a video connection and the ability to create and digitally deliver medication prescriptions and certificates were also highlighted as important needs. Indeed, delivering care mostly ends up in a prescription, a referral or a medical leave or other certificate [10, 15].

In the particular Belgium context teleconsultations and by extension telemedicine are not very commonly used yet. There is a provisional legal and financial framework but there are no generally implemented practice guidelines. This means that triage doctors are less competent in handling teleconsultations as compared to doctors working in countries where teleconsultations are more commonly used [9, 10, 15]. Above, patients are not confident at all with this type of clinical encounter and do not consider teleconsultations as a valuable patient-doctor contact. This might imply that the results presented in our study are an actual underestimation of the potential of teleconsultations.

### Strength and weakness

The major strength of the study is the high number of teleconsultations performed in a regular care situation with a neglectable number of drop out. Another strength is the multidisciplinary assessment of the teleconsultations by GPs, medical specialists and a mix of young and experienced doctors.

The major weakness is the lack of a qualitative deepening of the data. The data were mainly collected in a quantitative way since this approach interfered the least with the regular care. Second, we did not compare the outcome of cases as assessed by triage (tele-) doctors and the doctors who examined the patients. There was no confirmation of diagnosis or fitness of referral by triage doctors and we only relied on the interpretation of the triage doctors. In a safety and effectiveness study, assessment of cases should be compared.

## Conclusion

Teleconsultations in unplanned primary care can be performed with a high quality and a sufficient level of certainty. The willingness to conduct teleconsultations in unplanned care is high. Despite this, a considerable proportion of teleconsultations are currently perceived as insufficient to provide care. Others, face-to-face contact with the patient is not highly missed which might imply that supportive protocols to triage patients to teleconsultations are necessary. Communication and technical problems appeared no important barriers.

To make teleconsultations feasible in the future, there is a need for clear protocols describing the nature of a teleconsultation. The option to create medication prescriptions and certificates and deliver them to the patient should also be added to the teleconsultation. In addition, there is a need for the availability of a video connection in the particular cases where a visualization of the clinical problem is required.

It would be useful in a future study to investigate the feasibility, obstacles and needs for implementation of video consultations as they may differ from teleconsultations. Also the inclusion and exclusion criteria for the performance of tele- and/or video consultations must be established by means of further research.

## Data Availability

Data are available at https://kuleuven-my.sharepoint.com/:x:/g/personal/birgitte_schoenmakers_kuleuven_be/EWJBaNh3HZdEkislpl2jFFkBMs0v0QgApQoat5mI90XZdw?e=PCclyM. https://kuleuven-my.sharepoint.com/:x:/g/personal/birgitte_schoenmakers_kuleuven_be/EWlj_xtnyxxHo5YClkBvfFsBbfup5YoCFrE14CJzRQvc_g?e=xWxsz2.

## Attachment 1: survey questions

On a scale from 1-100

1. What do you think was the quality of the consultation?
2. What was the quality of the contact with the patient during the consultation?
3. To what extent did you miss face-to-face contact with the patient?
4. To what extent did you experience communication problems with the patient?
5. To what extent did you experience technical problems?
6. What was the degree of certainty with which you were able to complete this consultation?
7. To what extent could a physical examination have contributed to the diagnosis in this consultation?
8. To what extent could video have contributed to the diagnosis in this consultation?
9. To what extent are you prepared to make use of teleconsultations during on-call duty in the future?

## List of abbreviations

KCE: Federal Knowledge Center for Healthcare
GP: General practitioner
MUG: Mobile Emergency Group
PIT: Paramedical Intervention Team
HAIOs: Residents in general practice
ASOs: Hospital residents in emergency medicine
